# Metabolite Profiling and Bioactivity of Eastern Algerian *Posidonia oceanica*: Cholinesterase and Urease Inhibition with Low Cytotoxicity

**DOI:** 10.1021/acsomega.5c08586

**Published:** 2025-09-30

**Authors:** Malaoui Karima, Karouche Saida, Ouided Benslama, Hamdi Bendif, Sabrina Lekmine, Ramazan Erenler, Dib Mouna, Lamjed Bouslama, Sulaiman A Alsalamah, Tarek H. Taha, Fehmi Boufahja, Stefania Garzoli

**Affiliations:** † Laboratory of Natural Substances, Biomolecules, and Biotechnological Applications, Department of Natural and Life Sciences, 257318 Larbi Ben M’Hidi University, Oum El Bouaghi 04000, Algeria; ‡ Laboratory of Ecology and Animal Physiology, Department of Natural and Life Sciences, Larbi Ben M’Hidi University, Oum El Bouaghi 04000, Algeria; § Department of Biology, College of Science, 48024 Imam Mohammad Ibn Saud Islamic University (IMSIU), Riyadh 11623, Saudi Arabia; ∥ Biotechnology, Water, Environment and Health Laboratory, 271969 Abbes Laghrour University, Khenchela 40000, Algeria; ⊥ Research Laboratory Practice and Research Center, Igdir University, Igdir 7600, Türkiye; # Laboratory of Bioactive Substances-LR15CBBC03, 155529 University of Tunis El Manar, Center of Biotechnology of Borj Cedria, Hammam Lif 2050, Tunisia; ∇ Department of Chemistry and Technologies of Drug, Sapienza University, P. le Aldo Moro, 5, Rome 00185, Italy

## Abstract

Given the growing need for sustainable and natural alternatives to address global health and environmental challenges, marine plants have emerged as promising sources of bioactive compounds. In this context, the present study aimed to investigate the therapeutic potential of *Posidonia oceanica* (L.) Delile through chemical profiling and enzymatic inhibitory evaluation. Samples (leaves and rhizomes) were collected from the eastern Algerian coast in 2023. Methanolic extracts were prepared and analyzed using liquid chromatography coupled with tandem mass spectrometry (LC-MS/MS). The inhibitory activity of the extracts was evaluated against three key enzymes: urease, acetylcholinesterase (AChE), and butyrylcholinesterase (BChE), using Ellman’s colorimetric method for AChE and BChE, and the indophenol-based colorimetric method for urease. Cytotoxicity was assessed using the MTT colorimetric assay on Vero cells.LC-MS/MS analysis led to the identification of 23 secondary metabolites in the leaf extract and 22 compounds in the rhizome extract. The leaf extract exhibited an IC_50_ of 15.78 ± 0.34 μg/mL against urease, and 113.43 ± 0.77/30.34 ± 0.56 μg/mL against AChE and BChE, respectively. The rhizome extract showed IC_50_ values of 20.52 ± 0.75 μg/mL for urease and 33.59 ± 0.73/11.82 ± 0.73 μg/mL for AChE and BChE, respectively, demonstrating comparable or even superior activity to reference inhibitors such as thiourea and galantamine. Cytotoxicity results revealed moderate toxicity only at high concentrations, while the effective doses used in enzymatic assays were well below the toxicity threshold. Moreover, the selectivity index (SI) highlighted the promising potential of the rhizome extract against BChE (≈24.61) and of the leaf extract against urease (≈21.10), which strengthens their balance between efficacy and biological safety. Molecular docking studies further confirmed these results, showing strong binding interactions between phenolic compounds and the active sites of target enzymes. Notably, polydatin showed strong affinity for AChE (−7.765 kcal/mol), closely matching galantamine; rutin exhibited the highest binding energy with BChE (−9.533 kcal/mol); and vanillin demonstrated the best binding affinity for urease (−5.235 kcal/mol), surpassing thiourea. The phenolic compounds in *P. oceanica* exhibited promising multitarget inhibitory activity, particularly in the medical field for treating neurodegenerative diseases and urease-related infections. The observed moderate cytotoxicity indicates an acceptable safety margin, enhancing their value as a natural source for applications in medical, veterinary, agricultural, and environmental fields.

## Graphical abstract

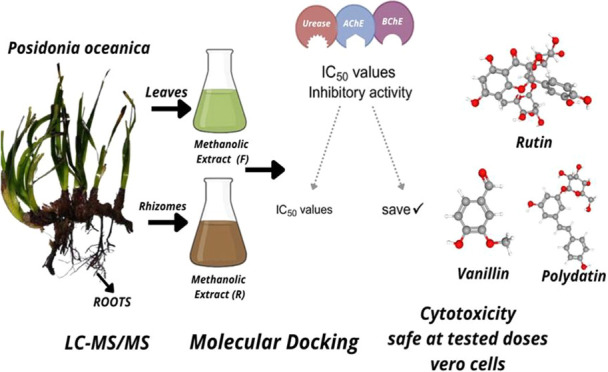

## Introduction

The marine environment harbors extraordinary biodiversity and represents an invaluable yet underexplored reservoir of bioactive natural compounds with significant pharmacological potential. Marine organisms, ranging from microorganisms to macroalgae and seagrasses, have evolved complex biochemical systems to survive extreme oceanic conditions—resulting in the production of unique metabolites with anti-inflammatory, anticancer, antiviral, and neuroactive properties. Compounds such as saxitoxin, tetrodotoxin, and curacin-A have demonstrated potent effects on ion channels and neuronal enzymes, offering critical insight into the development of neuropharmacological agents, including those for neurodegenerative diseases like Alzheimer’s.^1,2^

Among these marine resources, *Posidonia oceanica* is a long-lived seagrass species endemic to the Mediterranean and plays a crucial ecological role as an “ecosystem engineer” and a major reservoir of “blue carbon.” Its meadows provide essential habitats and nursery grounds for numerous marine organisms, stabilize sediments, and significantly contribute to carbon sequestration, thereby enhancing biodiversity and supporting climate regulation in the Mediterranean basin. This recently gained considerable scientific attention due to its broad spectrum of pharmacological properties. Previous studies have demonstrated that extracts from *P. oceanica* exhibit antioxidant, anti-inflammatory, and antimigratory effects, particularly through the inhibition of neuroblastoma cell migration and the induction of autophagy—highlighting its potential as a multifunctional neuroprotective agent.^3^ Moreover, recent advancements in drug delivery technologies have shown that nanoformulations of *P. oceanica* leaf extract, such as chitosan nanoparticles and Soluplus polymeric micelles, significantly improve its aqueous solubility and enhance its antimigratory bioactivity. These findings support the plant’s potential as a technologically adaptable marine phytocomplex for therapeutic applications.^4^

In a different context, Alzheimer’s disease (AD) is one of the most prevalent neurodegenerative disorders associated with aging. In 2021, Alzheimer’s disease and other forms of dementia ranked as the seventh leading cause of death, killing 1.8 million lives. Women are disproportionately affected. Globally, 68% of deaths from Alzheimer’s and other forms of dementia are women.^5^ Projections indicate that the number of people affected by dementia is expected to reach 152.8 million by 2050,^6^ representing a 4-fold increase. It is characterized by progressive memory loss and cognitive decline due to a decrease in the level of acetylcholine, a key neurotransmitter in the brain.^7,8^ For decades, treatment strategies have focused primarily on inhibiting acetylcholinesterase (AChE), the enzyme responsable for the breakdown of acetylcholine, thereby increasing its availability and improving cholinergic transmission.^7^

However, recent studies have emphasized the growing importance of butyrylcholinesterase (BChE), particularly in the later stages of AD, where AChE activity decreases while BChE activity increases in brain tissue.^9,10^ Selective BChE inhibitors have emerged as promising therapeutic agents to mitigate cognitive decline. Advanced research strategies—including molecular modeling, computer-aided drug design, and structure-based drug discovery—have yielded novel inhibitors with high selectivity and potency.^11,12^ Some of these compounds have demonstrated enhanced memory and cognitive performance in animal models.^12^ The development of both selective BChE inhibitors and dual AChE/BChE inhibitors is expected to improve our understanding of AD pathogenesis and lead to more effective treatment options.^9,13^

In this context, natural products—particularly those derived from marine organisms—have drawn increasing attention due to their chemical diversity, biological activity, and relatively low toxicity compared to synthetic drugs.^14^ Various marine species, especially those from the genera *Ecklonia*, *Sargassum*, and *Gracilaria*, have shown promising cholinesterase inhibitory activity. However, limited attention has been given to other marine phanerogams such as *P. oceanica*, despite its ecological significance and initial pharmacological potential.^2,15^

In addition to acetylcholinesterase inhibition in the medical field, urease inhibition emerges as another highly relevant target for natural products, owing to the enzyme’s involvement in diverse medical, agricultural, and environmental problems. Urease, a dinickel metalloenzyme (EC 3.5.1.5), plays a central role in catalyzing the hydrolysis of urea into ammonia and carbonic acid.^16,17^ Playing an essential role in the nitrogen cycle. Despite its biological importance, urease activity has been associated with several harmful effects across different fields.^18^

In the medical context, urease is recognized as a key virulence factor in pathogenic bacteria such as *Helicobacter pylori*, *Proteus mirabilis*, and *Klebsiella pneumoniae*. These microorganisms exploit urease to produce ammonia, which raises the local pH, disrupts host defense mechanisms, and facilitates bacterial colonization, especially in acidic environments like the stomach. The resulting ammonia production is associated with the development of peptic ulcers, gastritis, gastric cancer.^19^ Beyond the gastrointestinal tract, urease is implicated in urinary tract infections, kidney stone formation, hepatic encephalopathy, and catheter encrustation, reinforcing its role as a virulence factor in diverse clinical conditions.^20,21^

In the agricultural sector, urease plays a major role in reducing the efficiency of urea-based fertilizers. Upon application to soil, urea is rapidly hydrolyzed by urease enzymes present in soil and manure, resulting in the loss of nitrogen through ammonia volatilization. This process not only decreases fertilizer efficiency and increases production costs but also contributes to environmental degradation by elevating soil pH and promoting air and water pollution.^22,23^

Moreover, urease plays a detrimental role in animal production, especially in poultry and livestock farming, where its activity in the gastrointestinal tract or litter results in excessive ammonia emissions.^24,25^ This leads to poor air quality, respiratory irritation, reduced feed conversion rates, weakened immune response, and increased mortality in animals.^26^

To counter these negative impacts, various synthetic urease inhibitors such as *N*-(*n*-butyl) thiophosphoric triamide (NBPT) and thiourea derivatives have been developed. These compounds work by chelating nickel ions at the enzyme’s active site or modifying essential cysteine residues, showing considerable effectiveness in both medical and agricultural applications. However, their use raises concerns regarding toxicity, chemical instability, environmental persistence and potential for antimicrobial resistance.^27–29^

In response, there is growing interest in natural urease inhibitors derived from plants. Compounds such as polyphenols, flavonoids, and tannins from sources have demonstrated urease inhibitory activity with lower toxicity and higher biocompatibility. Additionally, structure–activity relationship (SAR) analyses and molecular docking techniques have facilitated the rational design of novel urease inhibitors with improved efficacy and specificity. Despite the progress made, the search for low-toxicity, stable, and environmentally safe urease inhibitors remains a pressing challenge.^30^

Based on these premises, this study aims to conduct a comprehensive evaluation of the inhibitory activity of *P. oceanica* extracts against three key enzymes: urease, acetylcholinesterase (AChE), and butyrylcholinesterase (BChE). To achieve this, an integrated approach was adopted, combining *in vitro* biochemical assays, compound identification through liquid chromatography-tandem mass spectrometry (LC-MS/MS), and molecular docking studies to elucidate the structural mechanisms of inhibition. A cytotoxicity assessment was also performed to evaluate the safety and biological compatibility of the extracts for potential therapeutic use. Through this multidisciplinary framework, the study seeks to highlight the pharmacological and biotechnological potential of *P. oceanica* as a natural, safe, and sustainable source of multitarget enzyme inhibitors, in comparison with established reference compounds, and within the broader context of innovative applications in medicine, neurology, agriculture, and veterinary science.

## Results and Discussion

### Chemical Composition of *Posidonia oceanica*

The qualitative and quantitative characterization of phenolic compounds in *P. oceanica* was performed using LC-MS/MS, based on a panel of 43 reference standards. Twenty-three compounds were identified in the leaf extracts (FCJ, FCN, FSN) and 22 in the rhizome extracts (RCJ, RCN, RSN), with 3 compounds being exclusive to either tissue (Table 1 and Figure 1). It is noteworthy that these variations may be attributed to a combination of associated or independent factors such as collection period and geographical location.

**Figure 1:**
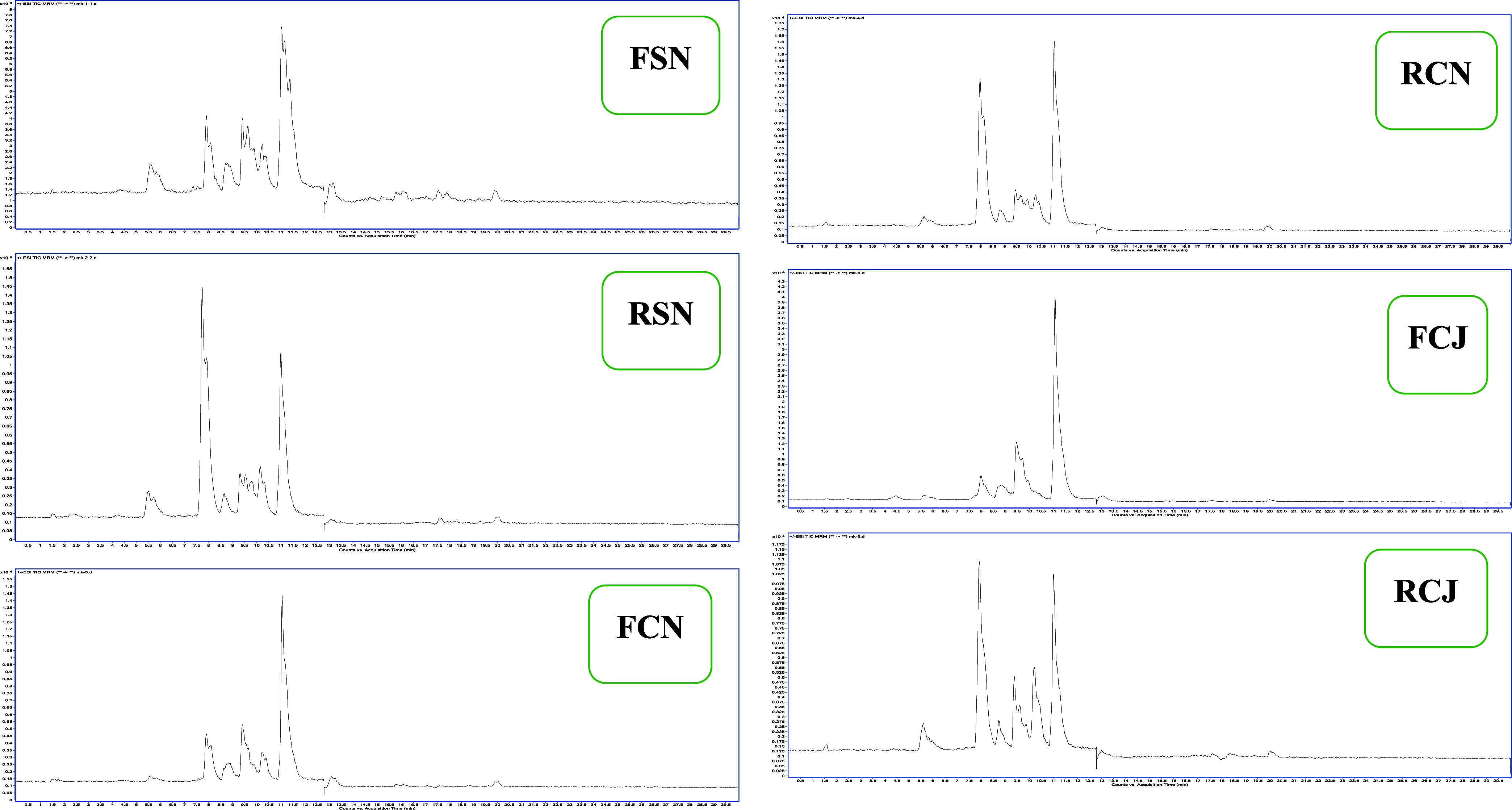
LC-MS-MS chromatograms of chemical compounds from different extracts of *P. oceanic*.

**Table 1:** Quantitative Analysis of Natural Compounds in *P. oceanica* by LC-MS/MS (μg/g Extract)^a^

quantitation results							
compound	RT	FCJ	FCN	FSN	RCJ	RCN	RSN
shikimic acid	2,017967	ND	ND	546,4074062	ND	ND	ND
gallic acid	4,476683	ND	ND	4,627332922	ND	ND	4,717480018
protocatechuic acid	5,429533	45,5888352	36,60494005	40,284277	97,82745723	47,8857528	125,9810932
hydroxybenzaldehyde	8,56725	ND	0,421652577	ND	9,396755006	3,51634355	3063,916802
epigallocatechin	8,602567	ND	ND	ND	ND	ND	ND
catechin	8,6719	817,405775	ND	11,14786128	4114,99436	4271,70054	9,94563137
chlorogenic acid	8,812167	ND	ND	ND	ND	ND	ND
caffeic acid	9,442867	205,165186	77,65055927	37,45612359	15,17148765	13,8694685	23,63856115
vanillic acid	9,542883	813,707878	591,4455181	ND	2222,847303	3332,87824	ND
syringic acid	9,682233	ND	ND	ND	ND	ND	ND
caffeine	9,8702	ND	ND	ND	ND	ND	ND
vanillin	9,299267	23,0066079	36,93282785	ND	44,72482486	31,3160921	ND
resveratrol	10,17602	ND	ND	84,75354344	ND	ND	784,5309632
polydatin	10,20822	1,02987877	ND	ND	1,606358859	ND	ND
salicylic acid	10,14807	37,4330944	95,40078743	ND	245,1833705	115,80804	2,048718468
*p*-coumaric acid	9,816283	503,529094	47,05342229	ND	42,75604652	86,7650971	0,181230834
taxifolin	10,46552	ND	ND	256,5702744	ND	ND	83,24568325
sinapic acid	10,84917	ND	ND	ND	ND	ND	ND
trans-ferulic acid	10,65923	1010,68295	71,79910182	ND	ND	99,038263	ND
scutellarin	10,80398	ND	ND	88,51495799	ND	ND	155,1207602
hesperidin	11,25007	ND	ND	ND	ND	ND	ND
rutin	11,1673	ND	ND	155,6773805	ND	31,9191664	21,81354365
isoquercitrin	11,02727	6670,05706	2362,620261	ND	1497,908684	2714,80738	ND
*o*-coumaric acid	11,51322	7,95841006	ND	ND	1,029469441	62,4030163	ND
coumarin	11,12887	23,3984244	12,4404583	ND	15,8189753	5,663128	ND
protocatehuic ethyl ester	11,66935	ND	ND	ND	ND	ND	ND
quercetin-3-d-xyloside	12,32998	ND	ND	ND	ND	ND	0,862056485
kaempferol-3-glucoside	13,12227	ND	ND	ND	ND	ND	ND
naringenin	13,1213	26,3883239	19,09416998	ND	10,36273167	7,69290399	ND
trans-cinnamic acid	13,0173	12,8923799	24,44104542	ND	ND	6,86967175	ND
morin	13,41933	ND	ND	ND	ND	ND	ND
quercetin	13,40703	86,3576803	34,35346902	ND	26,55257219	20,4675606	ND
hesperetin	13,4573	ND	ND	ND	ND	ND	ND
baicalin	13,67097	ND	ND	ND	ND	ND	ND
fisetin	13,87523	ND	ND	352,606454	ND	ND	ND
kaempferol	15,25523	ND	ND	ND	ND	ND	ND
baicalein	14,56	ND	ND	ND	ND	ND	ND
luteolin	15,52097	ND	ND	ND	ND	ND	ND
biochanin A	15,3273	ND	ND	ND	ND	ND	ND
chrysin	15,40395	ND	ND	ND	ND	ND	ND
capsaicin	15,28332	ND	ND	0	ND	ND	0
dihydrocapcaicin	16,1603	ND	ND	0	ND	ND	0
diosgenin	23,43172	ND	ND	0	ND	ND	0

a ND: not detected, RT: retention time.

Comparative profiling revealed tissue-specific phenolic patterns: In the leaf extracts, isoquercitrin was the dominant compound in FCJ (6670.06 μg/g) and FCN (2362.62 μg/g), accounting for over 60% of total phenolics in FCJ. FSN was distinguished by the exclusive presence of flavonoids like taxifolin, rutin, and scutellarin. Conversely, RSN exhibited high levels of hydroxybenzaldehyde (3063.9 μg/g) and fisetin (352.6 μg/g), along with the presence of resveratrol (784.53 μg/g), not detected in leaf extracts. Both RCJ and RCN contained high catechin levels (4115 and 4271.7 μg/g, respectively), while RCN was the only extract to include trans-ferulic acid (99.04 μg/g) and o-coumaric acid (62.4 μg/g). These results highlight a clear tissue-specific differentiation in phenolic composition, with leaf extracts rich in common flavonoids and root extracts showing a unique profile enriched in specialized bioactive compounds.

### Inhibition of Enzymatic Activity

#### Inhibition of Cholinesterase Activity

The cholinesterase inhibitory activity of two *P. oceanica* extracts was assessed against acetylcholinesterase (AChE) and butyrylcholinesterase (BChE) enzymes by determining their half-maximal inhibitory concentration (IC_50_) values in μg/mL Table 2.

**Table 2:** Evaluation of Enzymatic and Cytotoxic Activities (IC_50_ and CC_50_, μg/mL) of Methanolic Leaf and Rhizome Extracts of *P. oceanic* ^a^,^b^

extracts	yield (%)	urease inhibitory assay IC_50_ (μg/mL)	acetylcholinesterase inhibitory activity IC_50_ (μg/mL)	butyrylcholinesterase inhibitory activity IC_50_ (μg/mL)	cytotoxicity assay on Vero Cells CC_50_ (μg/mL)	SI (urease)	SI (AChE)	SI (BChE)
F	15.64	15.78 ± 0.34^b^	113.43 ± 0.77^a^	30.34 ± 0.56^b^	333 ± 44.19^a^	21.10	2.93	10.97
R	16.71	20.52 ± 0.75^a^	33.59 ± 0.73^b^	11.82 ± 0.73^c^	291 ± 13.45^a^	14.18	8.66	24.61
thiourea^c^	/	11.57 ± 0.68^c^	NT	NT	NT	/	/	/
galantamine^c^	/	NT	6.27 ± 1.15^c^	34.75 ± 1.99^a^	NT	/	/	/

a R: Rhizome, F: Leaves.

b IC_50_ values are defined as the concentration of 50% inhibition percentages, CC_50_: 50% cytotoxic concentration. The CC_50_ values are expressed in μg/mL; The assays were performed on Vero cells using the colorimetric MTT Method. IC_50_ and CC_50_ were calculated by linear regression analysis and expressed as the mean ± SD (*n* = 3). The values with different superscripts (a, b and c) in the same line are significantly different (*p* < 0.001). NT: non tested. SI: selectivity index (SI = CC_50_/IC_50_).

c Reference compounds.

For AChE inhibition, Extract R exhibited the highest activity, with an IC_50_ value of 33.59 ± 0.73 μg/mL, followed by Extract F at 113.43 ± 0.77 μg/mL. The reference compound, galantamine, showed superior inhibition with an IC_50_ of 6.27 ± 1.15 μg/mL. Although both extracts were less potent than the standard, Extract R demonstrated a moderate level of inhibition, suggesting the presence of bioactive constituents with selective affinity for AChE. In contrast, a different trend was observed in the inhibition of BChE. Extract R again showed the strongest effect, achieving an IC_50_ of 11.82 ± 0.73 μg/mL, followed by Extract F at 30.34 ± 0.56 μg/mL. Interestingly, both extracts outperformed the reference compound (galantamine), which recorded an IC_50_ of 34.75 ± 1.99 μg/mL.

#### Inhibition of Urease Activity

The urease inhibitory activity of *P. oceanica* (FCJ and RCJ) extracts was assessed by measuring the percentage of inhibition and calculating the corresponding IC_50_ values, with thiourea as the reference inhibitor Table 2. The FCJ extract demonstrated significant inhibitory activity, with an IC_50_ value of 15.78 ± 0.34 μg/mL, while the RCJ extract showed an IC_50_ of 20.52 ± 0.75 μg/mL. Comparatively, the IC_50_ of thiourea was 11.57 ± 0.68 μg/mL, indicating that both extracts exhibited considerable urease inhibition, though slightly less potent than the reference compound.

### Cytotoxicity Assay

The results of the cytotoxicity assay of *P. oceanica* extracts under investigation are presented in the Table 2. The 50% cytotoxic concentration (CC_50_) values for extracts rhizomes (R) and F leaves (F) were 291 ± 13.45 and 333 ± 44.19 μg/mL, respectively. It is noteworthy that both phenolic extracts exhibited comparable cytotoxic effects, with the toxicity threshold observed at 350 μg/mL.

The extraction yields of the leaf (16.71%) and rhizome (15.64%) extracts indicate that both plant organs provide a substantial amount of extractable material under the employed maceration conditions. The slightly higher yield from leaves may reflect their higher content of soluble metabolites or a more efficient solvent penetration. In contrast, the rhizomes, despite a slightly lower yield, are known to accumulate concentrated secondary metabolites, which can exhibit potent biological activities. From an agricultural perspective, these yields suggest that both leaves and rhizomes could be considered for further applications, such as natural fertilizers, since the extractable fraction is sufficiently high to justify practical utilization. Reporting and discussing these yields is crucial for assessing the feasibility of scaling up, standardizing bioactivity measurements per gram of starting material, and guiding potential industrial, pharmacological, or agricultural applications.

These extraction yield results are further supported by LC-MS/MS analysis of phenolic and flavonoid compounds in the methanolic extracts of leaves and rhizomes of the marine plant *P. oceanica* revealed a chemically diverse profile strongly influenced by both the season (summer/July and autumn/November) and the geographical collection site (A or B). A total of 24 bioactive metabolites were identified, many of which are associated with antioxidant, anti-inflammatory, antimicrobial, and anticancer activities.^31–35^

The detected concentrations varied considerably depending on environmental conditions, suggesting that factors such as sunlight exposure, salinity, and temperature affect the biosynthesis of secondary metabolites.^36,37^ For instance, catechin was abundantly accumulated in the rhizomes from site A during both summer and autumn (4114 and 4271 μg/g, respectively), while nearly undetectable in autumn leaf samples. This stable accumulation indicates that the rhizome serves as a metabolic storage site for catechin, a well-known antioxidant and antimicrobial agent.^38–41^ The presence of catechin in Posidonia oceanica has been documented in most previous studies, which supports the present finding.^34,42^

Isoquercitrin showed a peak concentration in summer leaf extracts from site A (6670 μg/g), followed by a marked decline in autumn (2362 μg/g), indicating a pronounced seasonal effect on its biosynthesis. Similarly, protocatechuic acid, an antioxidant and anti-inflammatory phenolic acid, was consistently present and reached its highest level in the rhizomes from site B during autumn (125 μg/g).^42,43^

Hydroxybenzaldehyde was specifically detected in significant quantities (3063 μg/g) in autumn rhizomes from site B and was absent or negligible in other samples. This localized expression may reflect site-specific environmental stress or unique biosynthetic pathways. Hydroxybenzaldehyde is known for its antimicrobial and radical scavenging activity, and its accumulation in rhizomes could contribute to the plant’s adaptive response mechanisms.^44–47^

Other important phenolic acids such as vanillic acid and caffeic acid were present in substantial amounts, particularly in rhizomes, suggesting that these underground organs are critical sites for phenolic compound storage and synthesis. Additionally, resveratrol and polydatin were exclusively detected in autumn leaves from site B, possibly linked to environmental stressors such as oxidative stimuli or microbial presence. These compounds are of pharmacological interest due to their roles in modulating inflammatory and oncogenic pathways.^36,37,48^

Uncommon metabolites like taxifolin, scutellarin, and fisetin were found only in site B samples collected in autumn. Fisetin, present at 352 μg/g in the leaves, is of particular note for its strong antioxidant properties and its role in inducing apoptosis in cancerous cells.^49,50^ In contrast, compounds like coumarin and naringenin were more prevalent in summer samples, possibly due to their role in seasonal photoprotection or stress mitigation.^51–53^ Coumarin is known for anticoagulant and antifungal effects, while naringenin exhibits hypolipidemic and antidiabetic activities.^54,55^

A clear distinction was observed between the chemical profiles of leaves and rhizomes. Rhizomes predominantly accumulated simple phenolic acids such as catechin and protocatechuic acid, while leaves were richer in glycosylated flavonoids like isoquercitrin and rutin. This reflects distinct metabolic functions between the above- and below-ground parts of the plant. This pattern is largely consistent with most previous studies on *P. oceanica*, which have reported a similar distribution of phenolic compounds between plant organs, supporting the idea of organ-specific metabolic and defensive roles.^36,39,40,45,46,56^ Furthermore, the observed variation in metabolite profiles between sites A and B suggests that environmental stressors and anthropogenic activities, such as coastal pollution, temperature fluctuations, and nutrient accumulation, may influence the biosynthesis and distribution of phenolic and flavonoid compounds in Posidonia oceanica. These changes in secondary metabolite production could affect the plant’s adaptive capacity, including defense against microbial agents, protection of tissues from oxidative stress, and regulation of ecological interactions. Therefore, assessing the impact of environmental conditions and human activities on bioactive metabolite production is crucial for a comprehensive understanding of the pharmacological potential and ecological functions of this marine species.

Amid growing concerns over environmental pollution, antimicrobial resistance, and the limited efficacy of conventional chemical agents, plant-derived bioactive compounds have emerged as promising inhibitors of key enzymes involved in agricultural, veterinary, and medical challenges. These natural compounds have garnered significant interest due to their diverse mechanisms of action, high biocompatibility, and reduced toxicity compared to synthetic counterparts. Among the most widely targeted enzymes by plant-based inhibitors are urease, which contributes to ammonia volatilization and nitrogen loss in soil and livestock systems, and Anticholinesterase (AChE and BChE), which is implicated in neurological disorders and pesticide toxicity.^57–60^

Accordingly, this discussion aims to evaluate the effectiveness of the plant extracts investigated in this study as inhibitors of these two enzymes, by comparing their observed activities with those reported in the literature and assessing their practical relevance across different sectors.

The current study demonstrated the potential of a methanolic extract from *P. oceanica* a natural urease inhibitor Table 2, showing a clear dose-dependent response were observed, closely approximating the reference inhibitor thiourea. These results suggest that the extract, particularly FCJ, holds promising urease-inhibitory capacity.

Several previous studies have reported urease inhibitory activity in both terrestrial and marine plant extracts, with inhibition levels comparable to or slightly lower than those observed in the present study. For instance, Amin et al.^57^reported significant activity in extracts derived from terrestrial medicinal plants. Similarly, Lee et al.^61^ associated moderate inhibition with presence phenolic compounds. Ghous et al. and Biglar et al. similarly found urease inhibition by various botanicals with IC_50_ values between 30 and 60 μg/mL.^58,62,63^ Compared to these, the FCJ extract in the present study demonstrated superior or equivalent activity. This suggests the presence of promising bioactive constituents with potential application in sustainable nitrogen management strategies. To the best of our knowledge, there have been no reports in the literature on the inhibition of urease activity by *P. oceanica*.

The extraction method also appears critical. Methanolic extracts, as used in this study, have been widely reported as more effective compared to aqueous or acetone-based methods.^64,65^ This consistency supports the solvent’s role in isolating bioactive compounds relevant for urease inhibition.

In agricultural contexts, the inhibition of soil urease plays a vital role in reducing nitrogen loss from urea-based fertilizers despite the widespread use of synthetic inhibitors such as *N*-(*n*-butyl) thiophosphoric triamide (NBPT) and phenylphosphorodiamidate (PPD),^27,66^ their safety profile has come under increasing scrutiny. Krogmeier et al.^67^ demonstrated that at higher application rates, these compounds led to toxic accumulation of urea in plant tissues, causing leaf-tip necrosis in wheat (*Triticum aestivum* L.) and sorghum (*Sorghum bicolor*). This phytotoxicity was directly linked to the suppression of soil urease activity, which interfered with natural urea hydrolysis and disrupted nitrogen metabolism in plants. Nascimento et al. and Krogmeier et al.^67,68^ reported that more than 50% of nitrogen applied as conventional urea can be lost as ammonia within a few weeks of application, mainly due to rapid urease activity under favorable environmental conditions. Their study demonstrated that industrial treatments such as boric acid and copper sulfate coatings were able to reduce ammonia volatilization by over 75%, highlighting their effectiveness in stabilizing urea. However, these synthetic strategies present several drawbacks, including the use of heavy metals such as copper and zinc, which may lead to long-term soil and water contamination. Additionally, excessive boron accumulation can negatively impact sensitive crops and disrupt soil chemistry. The high cost and limited accessibility of coated urea formulations also limit their applicability in low-input farming systems. These limitations reinforce the need to identify safe, cost-effective, and sustainable alternatives. In this context, the use of plant-derived urease inhibitors appears particularly promising, offering a biologically compatible, environmentally friendly solution that aligns with the principles of sustainable agriculture.^28,51,68–72^

As studies have shown, the use of plant extract-based biocoated urea effectively contributes to enhancing plant growth under various soil conditions, highlighting its practical value in improving agricultural productivity in a sustainable and environmentally friendly manner.^22,73–76^ These findings resonate with the inhibitory activity shown by *P. oceanica* extract and warrant further investigation into its agronomic application.

Urease inhibition also has significant medical and veterinary relevance. Bacteria such as *H. pylori* and *P. mirabilis* utilize urease to colonize host tissues and elevate pH through ammonia production, contributing to diseases such as peptic ulcers, urinary tract infections, and kidney stones.^57,58^ In poultry and livestock systems, microbial urease contributes to ammonia buildup in litter, leading to respiratory issues and reduced productivity.^77^ Bai et al.^78^ demonstrated that certain plant extracts, particularly the methanolic extracts of *Acacia nilotica*, *Terminalia chebula*, and *Emblica officinalis*, possess significant urease inhibitory activity. This activity plays a crucial role in reducing the pathogenicity of urease-producing bacteria implicated in urinary tract infections. Their study confirmed the efficacy of these extracts against key urease-positive pathogens such as *Staphylococcus aureus*, *Proteus vulgaris*, *K. pneumoniae*, and *Pseudomonas aeruginosa*, highlighting the potential of these plants as natural antimicrobial agents. The methanolic extract of *P. oceanica* exhibited clear urease-inhibitory activity, with a dose-dependent response comparable to that of standard inhibitors such as thiourea. These findings are consistent with previous reports on plant-derived urease inhibitors and suggest meaningful potential across agricultural and medical application.

Accordingly, natural extracts—including *P. oceanica*—can be considered as innovative sources of urease inhibitors. Based on this, we propose several practical applications of the studied extract: its incorporation into urea as a biocoating for fertilizer granules or addition during fertilizer production, with the optimization of dosage determined through laboratory and greenhouse trials; in poultry farming, administration through feed or drinking water, or direct application onto litter, with regular monitoring of moisture levels and periodic reapplication to sustain inhibition. These recommendations are consistent with previous literature on the use of plant extract-based coatings and urease inhibitors in animal production systems, and they strongly support the potential for field-scale implementation of natural extracts as effective and environmentally friendly urease inhibitors.

The present study demonstrated a strong inhibitory activity of *P. oceanica* extracts—specifically from the leaves and rhizomes—against acetylcholinesterase (AChE) and butyrylcholinesterase (BChE), two key enzymes implicated in Alzheimer’s disease (AD) Table 2. The leaf extract exhibited considerable inhibition of BChE, with an IC_50_ value close to that of galantamine, while the rhizome extract showed almost twice the inhibitory potency of the reference drug. As for AChE, the rhizome extract again showed greater efficacy, being approximately three times more effective than the leaf extract.

This especially relevant in the context of AD progression, where BChE activity increases while AChE activity declines in the later stages of the disease.^9,10^ Therefore, the strong BChE inhibition observed, particularly from the rhizomes, could offer enhanced therapeutic benefit in advanced stages of Alzheimer’s.

These findings are in line with those of Orhan et al.,^79^ which remains the only previous study to investigate the cholinesterase inhibitory activity of *P. oceanica*. That study also assessed the leaves and rhizomes and reported potent BChE inhibition, confirming the presence of active compounds throughout different parts of the plant. The consistency between both studies strengthens the evidence for *P. oceanica* as a neuroprotective species of marine origin.

The strong cholinesterase inhibition observed is likely attributable to the plant’s richness in polyphenols and flavonoids, which are well-documented for their inhibitory activity against AChE and BChE.^11,80^ Similar classes of bioactive compounds have been identified in other marine plant species with neuropharmacological potential.

Additionally, a recent review by Lins et aland Olasehinde et al. highlighted that marine organisms, particularly brown algae such as *Ecklonia cava* and *Sargassum wightii*, and red algae like *Gelidiella acerosa*, are rich sources of BChE inhibitors. Notably, phlorotannins such as dieckol and phlorofucofuroeckol-A isolated from *E. cava* showed IC_50_ values as low as 0.9–2.7 μM, even surpassing synthetic cholinesterase inhibitors like donepezil. Similarly, extracts from *G. acerosa* and *S. wightii* achieved BChE inhibition rates above 70% at comparable concentrations.^81,82^

These findings reinforce the pharmacological relevance of marine-derived BChE inhibitors and place *P. oceanica* among the promising candidates in this category. The consistent BChE inhibition across marine species further supports the central role of polyphenolic structures—particularly phlorotannins—in mediating cholinesterase inhibition.^79,83–85^

Lins et al. also emphasized that discrepancies across studies may result from differences in extraction protocols, compound purity, collection season, and geographical variation. Therefore, standardizing extraction methods in accordance with marine pharmacognosy practices would improve data comparability and further validate the neurotherapeutic potential of *P. oceanica*.^81^

The studied extracts derived from the leaves and rhizomes of *P. oceanica* have previously demonstrated significant inhibitory activity against key enzymes implicated in chronic diseases, including urease, which is associated with gastric ulcers, and the cholinesterases (AChE and BChE), both of which are targets in Alzheimer’s disease therapy. These promising bioactivities were observed at relatively low concentrations, highlighting the therapeutic potential of the extracts.

Following the promising enzyme–inhibitory effects demonstrated by *P. oceanica* leaf and rhizome extracts—targeting key enzymes involved in pathological, environmental, and industrial processes—it was essential to evaluate their biological safety on noncancerous cells. For this purpose, the MTT assay was performed using Vero cells (African green monkey kidney epithelial cells), a well-established in vitro model for assessing general cytotoxicity.

The results showed that the CC_50_ values for the leaf and rhizome extracts were 333 ± 44.19 and 291 ± 13.45 μg/mL, respectively. According to the toxicity scale proposed by Nguta et al.^86,87^ the leaf extract can be considered weakly toxic or nontoxic, while the rhizome extract is moderately cytotoxic. More importantly, the concentrations at which significant enzyme inhibition was observed were considerably lower than the CC_50_ values, indicating a favorable biological safety profile and supporting their potential for future development in therapeutic, industrial, and environmental applications.

To date, most studies involving *P. oceanica* have focused primarily on its antiproliferative effects against cancerous cell lines such as HeLa, MCF-7and LS174,^88–90^ while its effects on normal, healthy cells remain underexplored. In this context, the present study represents one of the first to evaluate the cytotoxic potential of *P. oceanica* extracts on nontumorigenic cells using the Vero cell model, thus contributing new insights into the cellular safety of this marine plant.

Furthermore, when comparing the present findings with cytotoxicity data reported for other marine macroalgae,^91^ such as *Ulva flexuosa* and *Padina spp.*, which exhibited higher toxicity on Vero cells (with IC_50_ values below 100 μg/mL and cell viability dropping below 30% at 100 μg/mL), the extracts of *P. oceanica* showed significantly lower cytotoxicity. This comparative analysis underscores the relatively low toxicological risk of *P. oceanica*, reinforcing its suitability for further investigation and application across pharmaceutical and biotechnological fields.

The selectivity index (SI = CC_50_/IC_50_) adds an additional layer of biological relevance to the cytotoxicity findings, as it provides an integrated measure that balances inhibitory efficacy with safety margins. According to the classification proposed by Nguta et al.,^86^ high SI values are considered strong indicators of therapeutic potential. In this regard, the rhizome extract displayed a particularly high SI against BChE (≈24.61), positioning it as a promising candidate in the management of neurodegenerative disorders such as Alzheimer’s and Parkinson’s disease, where selective cholinesterase inhibition is a well-established therapeutic approach. On the other hand, the leaf extract showed a favorable SI against urease (≈21.10), which underscores its dual relevance: medically, as a potential strategy to combat urease-related infections such as those caused by *Helicobacter pylori* and *Proteus* species; and in agricultural and veterinary applications, where urease inhibition can enhance nitrogen use efficiency and mitigate the environmental and health impacts of ammonia emissions. Importantly, such integration of enzyme inhibition profiles with SI values has not been previously reported for *P. oceanica*, highlighting the originality of our findings and their potential to open new avenues for both medical and nonmedical exploitation of these extracts.

In silico analysis has emerged as a powerful tool for drug discovery, enabling the identification of potential therapeutic compounds through computational approaches. Molecular docking simulations are particularly valuable for exploring the interactions between bioactive molecules and specific enzymatic targets, offering insights into binding affinities and interaction profiles.^63,92,93^ The enzymes acetylcholinesterase (AChE), butyrylcholinesterase (BChE), and urease play critical roles in various physiological and pathological processes, making them attractive therapeutic targets.

AChE is a key enzyme in cholinergic signaling, where its inhibition can help manage neurodegenerative conditions such as Alzheimer’s disease by increasing acetylcholine levels in the synaptic cleft. In this context, the docking of *P. oceanica* phenolic compounds against AChE was performed to explore their potential as natural inhibitors and to identify lead molecules with strong binding affinities and interaction profiles. The docking results for AChE, summarized in Table 3 and Figure 2, highlight the binding efficiencies and interaction profiles of the top five phenolic compounds from *P. oceanica*, compared with the cocrystallized ligand, galantamine. Galantamine, a known AChE inhibitor, served as the reference compound, exhibiting a binding energy of −7.960 kcal/mol. Its interactions with the enzyme included several strong hydrogen bonds with critical residues such as Tyr124, Ser125, and His447, as well as hydrophobic contacts with residues like Phe297 and Trp86, which are located within the active gorge of AChE. These interactions establish galantamine’s robust inhibitory effect on AChE.

**Figure 2:**
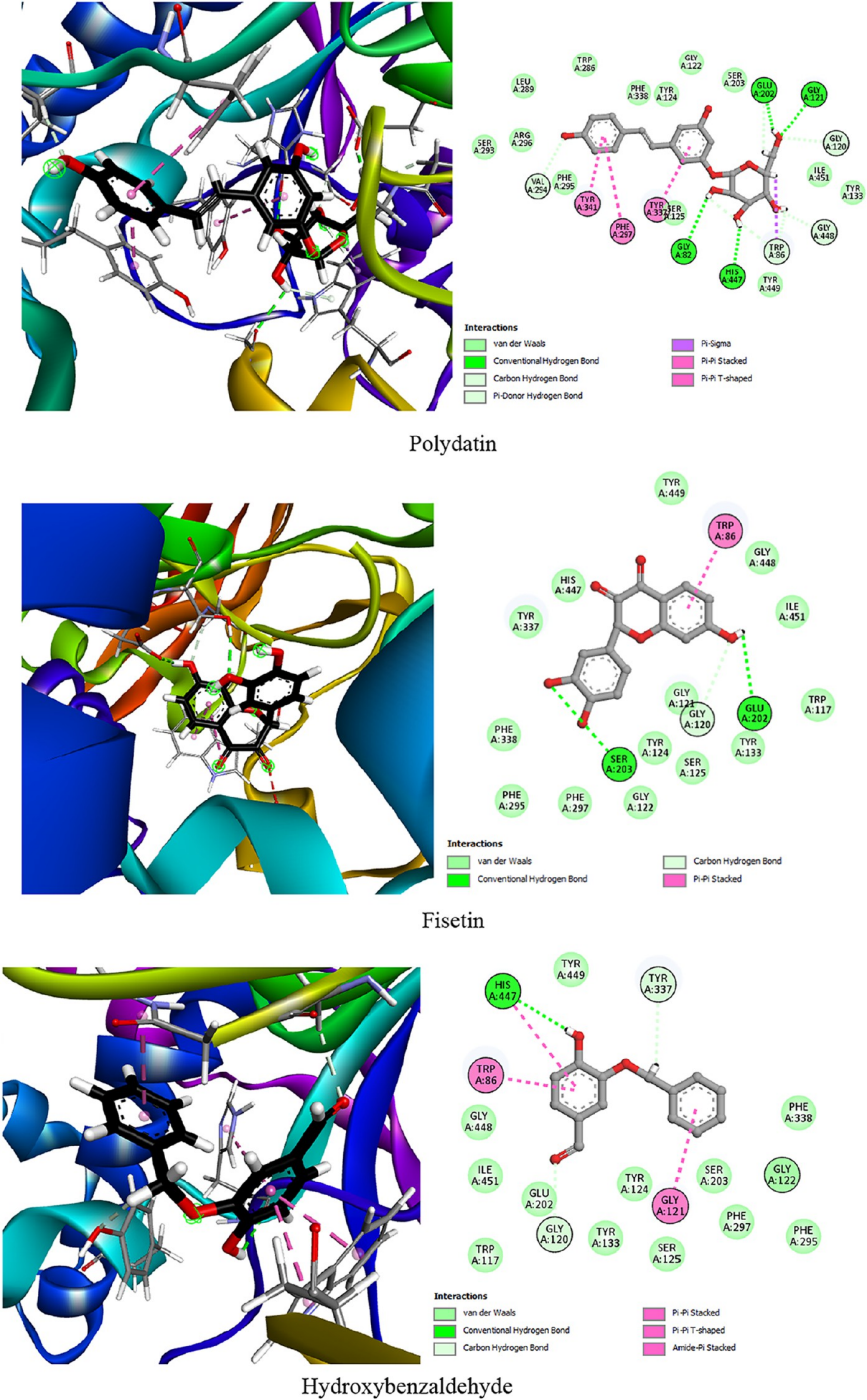

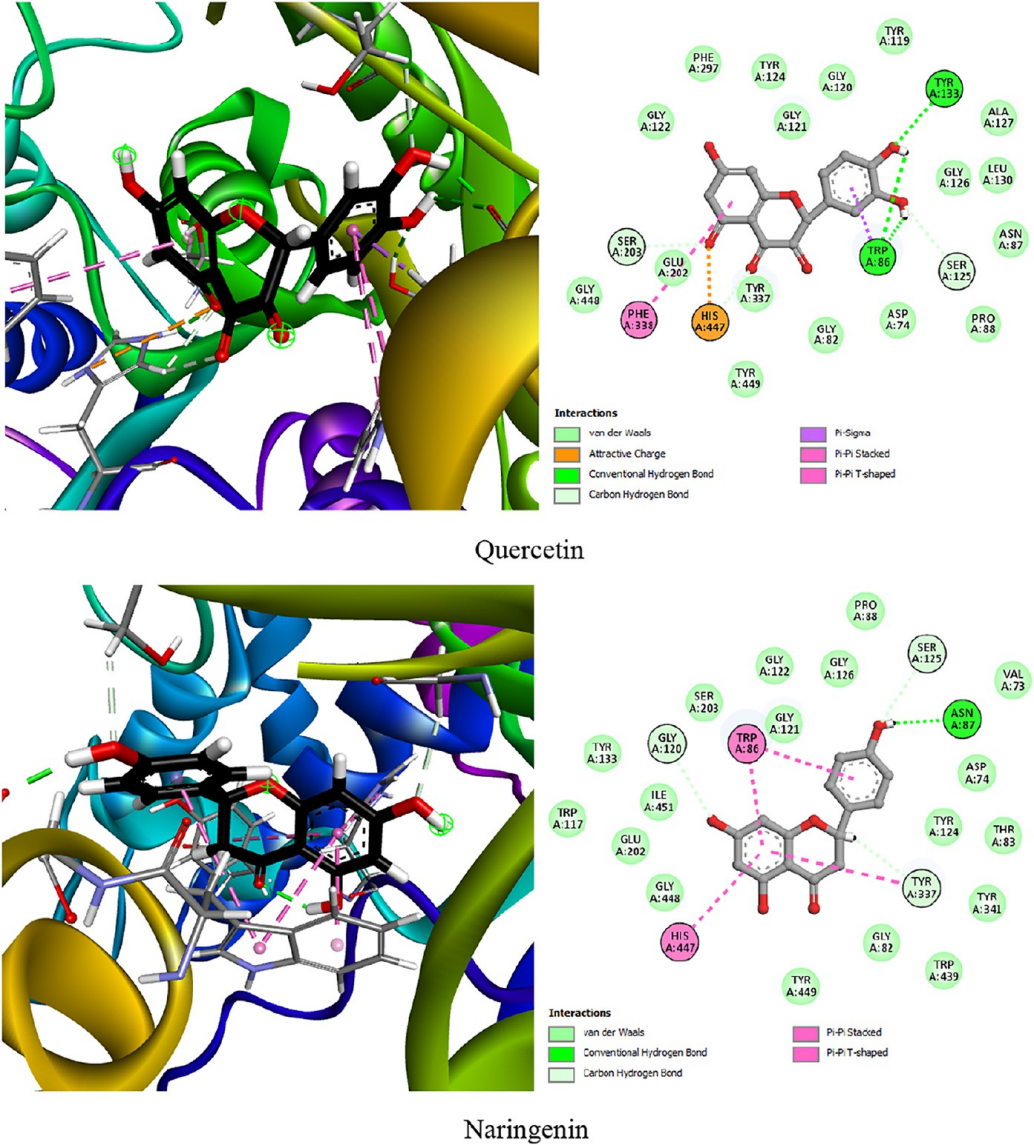
Two-dimensional (2D) and three-dimensional (3D) interaction diagrams of the top five *P. oceanica* phytocompounds within the active site of AChE. The 2D illustrations highlight hydrogen bonds, hydrophobic contacts, and electrostatic interactions with key active site residues, while the 3D visualizations provide a spatial representation of the ligand-enzyme binding.

**Table 3:** Best Five Results for the Docking of *P. oceanica* Phenolic Ligands with AChE Target

compound	binding energy (kcal/mol)	hydrogen interactions (distance Å)	hydrophobic interactions	electrostatic interaction
galantamine (co-crystallized ligand)	–7.960	Tyr124 (2.40), Tyr124 (2.52), Tyr337 (2.83) Ser125 (2.78) His447 (2.94), Ser203 (3.03), Gly121 (3.81) Glu202 (1.61) Gly120 (2.40), Gly82 (2.45) Asp74 (2.52)	Phe297, Trp236, Phe338, Phe295, His447, Gly121 Trp86	Asp74
polydatin	–7.765	Val294 (2.45), Gly82 (2.59), His447 (2.50), Trp86 (2.98), Trp86 (2.30), Gly448 (2.27), Gly120 (2.55), Gly121 (2.82), Glu202 (1.74), Glu202 (2.93)	Tyr341, Phe297, Tyr337, Trp86	-
fisetin	–7.333	Glu202 (2.61), Gly120 (2.44), Ser203(2.33)	Trp86	-
hydroxybenzaldeyde	–6.9792	Tyr337 (2.56), His447 (2.98), Gly120 (2.46)	Trp86, Gly121	-
quercetin	–6.723	Tyr133 (2.33), Trp86 (2.68), Trp86 (1.90), Ser125 (2.58), Ser203 (2.84), His447 (2.77)	Phe338	His447
naringenin	–6.565	Gly120 (2.58), Ser125 (2.28), Asn87 (1.73), Tyr337 (2.25)	Trp86, His447, Tyr337	-

Among the tested phenolic compounds, polydatin demonstrated the closest binding affinity to galantamine, with a binding energy of −7.765 kcal/mol. Polydatin formed multiple hydrogen bonds with residues Gly82, Gly120, Gly121, and Glu202, which are essential for stabilizing the ligand within the active site. Additionally, hydrophobic interactions with Tyr341, Phe297, and Tyr337 further reinforced its binding. The presence of these interactions indicates that polydatin could act as a competitive inhibitor of AChE, making it a promising candidate for further exploration.

Fisetin, with a binding energy of −7.333 kcal/mol, also showed strong interaction with key residues, including Glu202 and Gly120, through hydrogen bonding, while hydrophobic contacts with Trp86 contributed to its stabilization within the active site. Despite a slightly lower binding energy compared to polydatin, fisetin’s interaction profile suggests potential inhibitory activity against AChE.

Hydroxybenzaldehyde, quercetin, and naringenin demonstrated moderate binding affinities, with binding energies ranging from −6.565 to −6.979 kcal/mol. Hydroxybenzaldehyde formed hydrogen bonds with Tyr337 and His447 and hydrophobic interactions with Trp86 and Gly121, while quercetin interacted with Tyr133, Trp86, and Ser203. Notably, quercetin also formed electrostatic interactions with His447, which may enhance its binding affinity. Naringenin exhibited the weakest binding energy among the top compounds but formed hydrogen bonds with key residues such as Ser125 and Gly120, alongside hydrophobic interactions with Trp86 and Tyr337. While these compounds displayed slightly lower binding energies compared to galantamine, their interaction profiles suggest moderate inhibitory potential, warranting further evaluation.

The molecular docking results revealed that the *P. oceanica* phenolic compounds interact with key residues within the active site gorge of AChE, as highlighted in previous crystallographic studies. The conserved amino acids reported for AChE include catalytic and oxyanion hole residues (Ser203 and His447), choline-binding pocket residues (Glu202, Tyr337, and Trp86), and acyl-binding pocket residues (Trp236, Phe338, Phe297, and Phe295). These residues play critical roles in substrate recognition and catalytic efficiency, as supported by literature reports.^94,95^

In our study, the cocrystallized ligand galantamine formed interactions with several of these key residues, including Tyr124, His447, Ser203, and Gly121, confirming the reliability of the docking protocol. Similarly, the top-ranking phenolic compounds, such as polydatin and fisetin, established strong hydrogen bonds and hydrophobic contacts with residues like Glu202, Trp86, and Tyr337, which are essential for ligand stabilization within the active site. The ability of these compounds to interact with conserved residues highlights their potential as competitive inhibitors of AChE. This observation aligns with prior findings suggesting that interactions with these critical amino acids are essential for effective enzyme inhibition.^96^

The molecular docking results for the interaction of *P. oceanica* phenolic compounds with BChE provide valuable insights into their inhibitory potential. As summarized in Table 4 and illustrated in Figure 3, the docking analysis included two reference ligands: the cocrystallized ligand (3F9) and galantamine, a known BChE inhibitor used in our *in vitro* assays. These reference ligands served as benchmarks to validate the docking protocol and to compare the binding affinities and interactions of the *P. oceanica* phenolic compounds.

**Figure 3:**
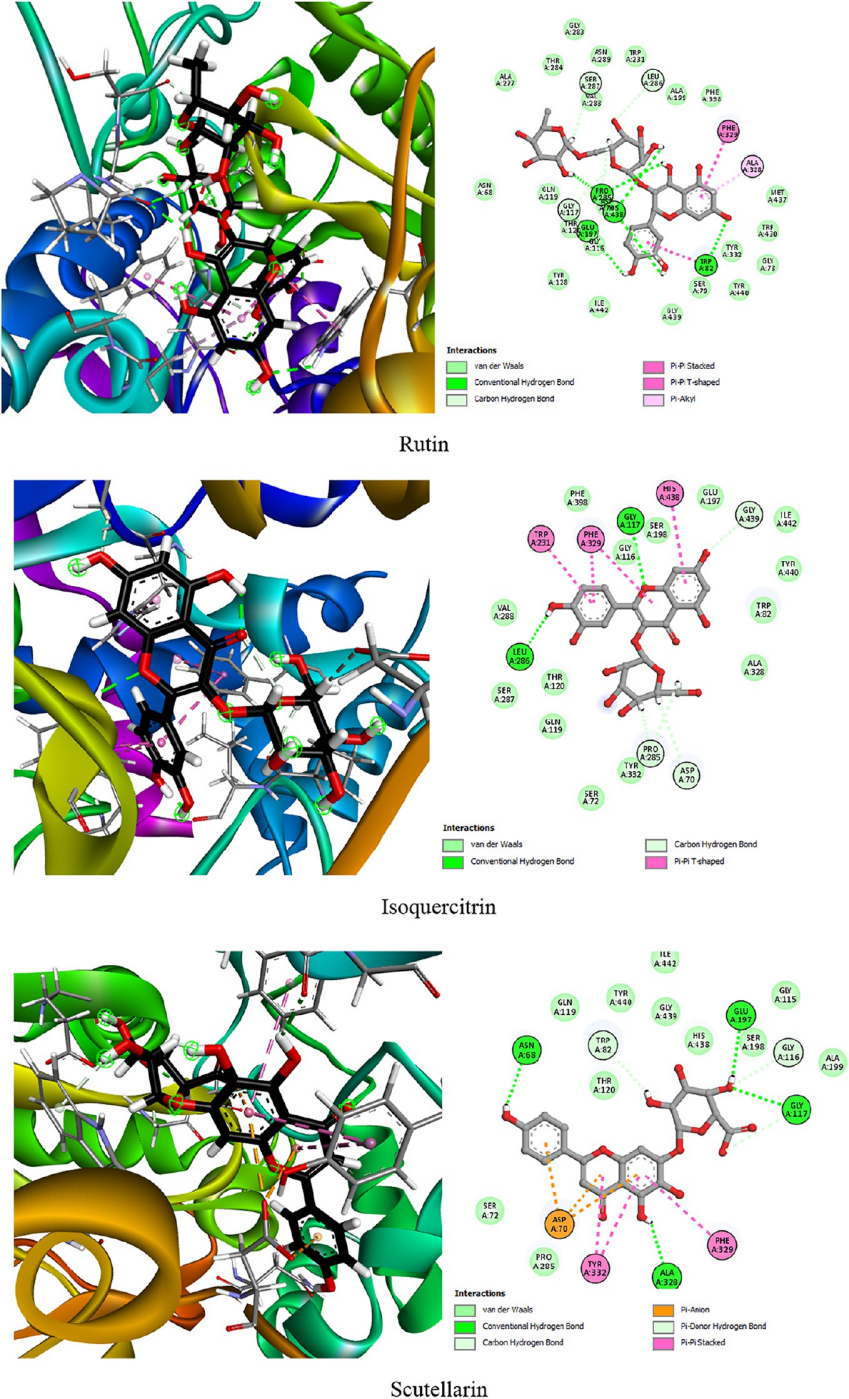

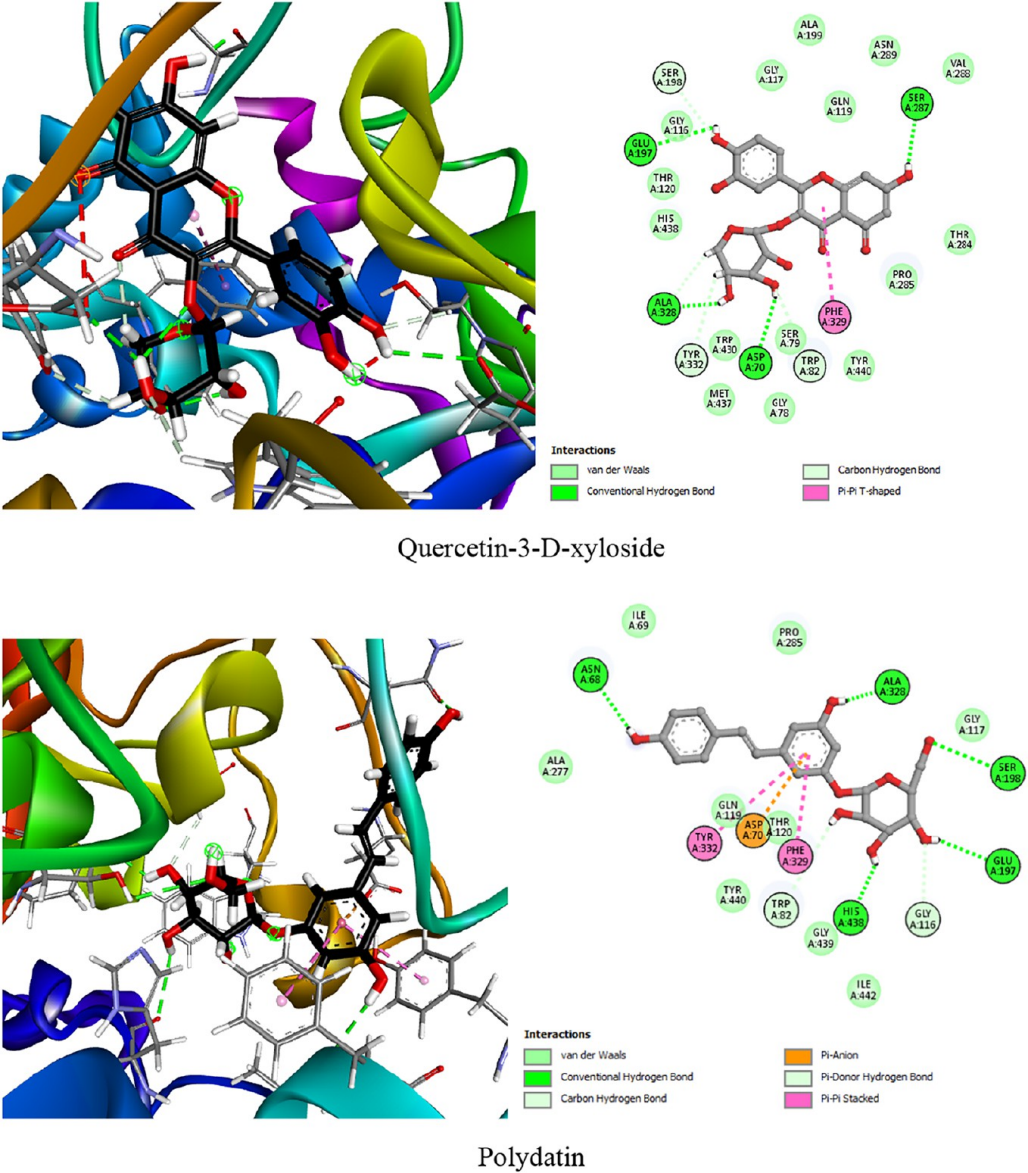
Two-dimensional (2D) and three-dimensional (3D) interaction diagrams of the top five *P. oceanica* phytocompounds within the active site of BChE. The 2D illustrations highlight hydrogen bonds, hydrophobic contacts, and electrostatic interactions with key active site residues, while the 3D visualizations provide a spatial representation of the ligand-enzyme binding.

**Table 4:** Best Five Results for the Docking of *P. oceanica* Phenolic Ligands with BChE Target

compound	binding energy (kcal/mol)	hydrogen interactions (distance Å)	hydrophobic interactions	electrostatic interaction
3F9 (co-crystallized ligand)	–8.521	Glu197 (2.49), Glu197 (2.66), Thr120 (2.50), Gly116 (1.75)	Gly116, Leu286, Phe329, Trp231	Asp70, Tyr332
galantamine	–6.521	Asp70 (2.22), Asp70 (2.70), Pro285 (2.39)	His438, Phe329, Trp82, Ala328	Asp70, Tyr332
rutin	–9.533	Ser287 (2.39), Leu286 (2.95), Trp82 (2.53), Gly197 (2.86) Gly117 (2.81) Pro285 (2.27), Pro285 (2.81), Pro285 (2.41), His438 (2.70), His438 (2.37), His438 (2.65)	Phe329, Ala328, Trp82	-
isoquercetin	–8.583	Gly439 (2.35), Gly117 (2.91), Leu286 (1.84), Pro285 (2.49), Pro285 (2.62), Asp70 (2.62)	His438, Phe329, Trp231	-
scutellarin	–7.688	Asn68 (1.97), Trp82 (2.55), Glu197 (2.38), Gly116 (2.83), Gly117 (2.49), Ala328 (2.16)	Phe329, Tyr332	Asp70
quercetin-3-d-xyloside	–7.678	Aer198 (2.96), Glu197 (2.80), Ala328 (2.35) Ala328 (1.96), Tyr332 (2.58) Asp70 (2.27), Trp82 (2.74), Ser287 (2.00)	Phe329	-
polydatin	–7.192	Asn68 (2.25), Ala328 (2.52), Ser198 (2.92), Glu197 (2.35), Gly116 (2.59), His 438 (2.99) Trp82 (2.65)	Phe329, Tyr332	Asp70

The cocrystallized ligand 3F9 exhibited the strongest binding affinity with a binding energy of −8.521 kcal/mol. It interacted with key catalytic site residues, including Glu197, Thr120, and Gly116, via hydrogen bonds, and established hydrophobic contacts with Leu286, Phe329, and Trp231. The presence of electrostatic interactions with Asp70 and Tyr332 further stabilized the ligand within the active site gorge. These results align with previous crystallographic data identifying these residues as critical for ligand binding and enzyme inhibition.

Galantamine, with a binding energy of −6.521 kcal/mol, demonstrated similar interactions with Asp70, Pro285, and Ala328, as well as hydrophobic contacts with His438, Phe329, and Trp82. The dual interactions with Asp70 highlight its importance as a catalytic residue, reinforcing the role of galantamine as a potent inhibitor of BChE. Although its binding energy was slightly lower than that of the cocrystallized ligand, its interactions validate its effectiveness as an inhibitor and justify its inclusion in the *in vitro* studies.

Among the *P. oceanica* phenolic compounds, rutin emerged as the top-ranking molecule, exhibiting the highest binding energy (−9.533 kcal/mol). Rutin formed a dense network of hydrogen bonds with key residues such as Ser287, Leu286, Trp82, Gly197, and His438. These interactions were complemented by hydrophobic contacts with Phe329 and Ala328, indicating a strong affinity for the BChE active site. Isoquercetin, another top compound, demonstrated a binding energy of −8.583 kcal/mol and shared critical interactions with Gly117, Leu286, and Pro285, as well as electrostatic contacts with Asp70.

The superior performance of rutin compared with its aglycone quercetin can be explained by the influence of glycosylation. The addition of a disaccharide moiety in rutin increases the number of hydrogen bond donors and acceptors, allowing the molecule to establish a dense interaction network with polar residues in the active site (e.g., Ser287, Gly197, His438). In contrast, quercetin, being smaller and less polar, forms fewer stabilizing contacts and tends to rely more on hydrophobic interactions. This structure–activity relationship suggests that glycosylation enhances binding stability by reinforcing ligand–protein interactions, despite the steric bulk it introduces. The same trend is observed for other glycosylated flavonoids such as isoquercetin and quercetin-3-D-xyloside, which also rank among the top inhibitors. Thus, the presence and position of sugar moieties emerge as critical determinants of binding affinity in this enzyme system.

Other notable compounds included scutellarin and quercetin-3-d-xyloside, which formed multiple hydrogen bonds with residues like Glu197, Gly116, and Ala328, and hydrophobic interactions with Phe329 and Trp82. Polydatin also exhibited significant interactions with Asn68, Ala328, and His438, underscoring the potential of phenolic compounds to act as competitive inhibitors of BChE.

The interactions of these compounds with conserved catalytic residues, such as Ser198, His438, Glu197, and Asp70, highlight their ability to target the active site effectively. These residues, well-documented in crystallographic studies, are pivotal for BChE activity and are key determinants of inhibitor binding. The ability of the *P. oceanica* phenolics to form stable interactions with these residues suggests that they could serve as promising leads for the development of novel BChE inhibitors.

Urease is a key enzyme involved in urea hydrolysis, playing a critical role in various pathological conditions, including infections caused by urease-producing bacteria. The inhibition of urease is therefore a promising therapeutic strategy, and thiourea is commonly used as a reference inhibitor for studying this enzyme.^97^ In this study, the molecular docking of *P. oceanica* phenolic ligands with urease was performed to evaluate their inhibitory potential. The results of this analysis, summarized in Table 5 and illustrated in Figure 4, provide insights into the binding affinities and interaction patterns of the best tested compounds in the active site of the enzyme.

**Figure 4:**
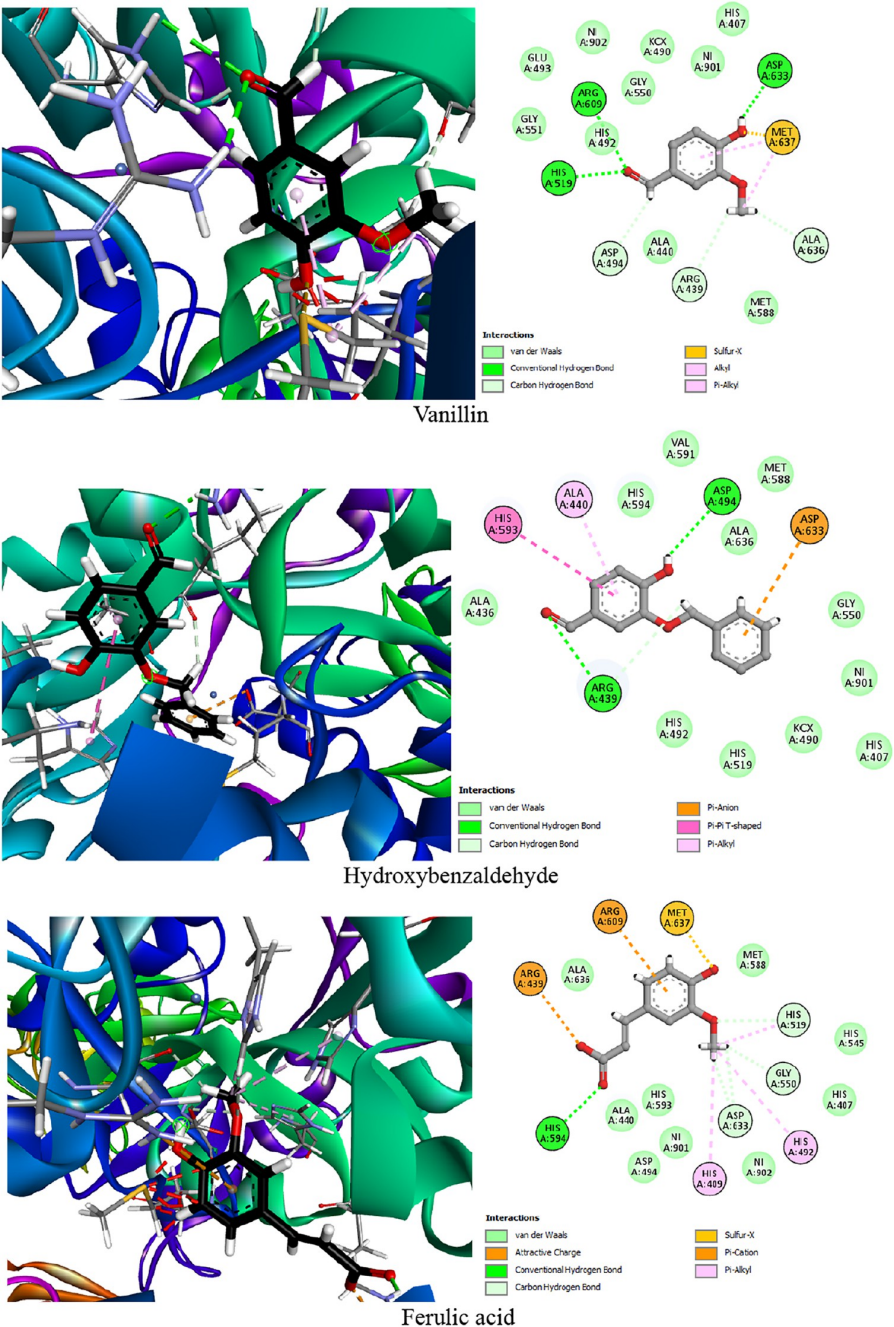

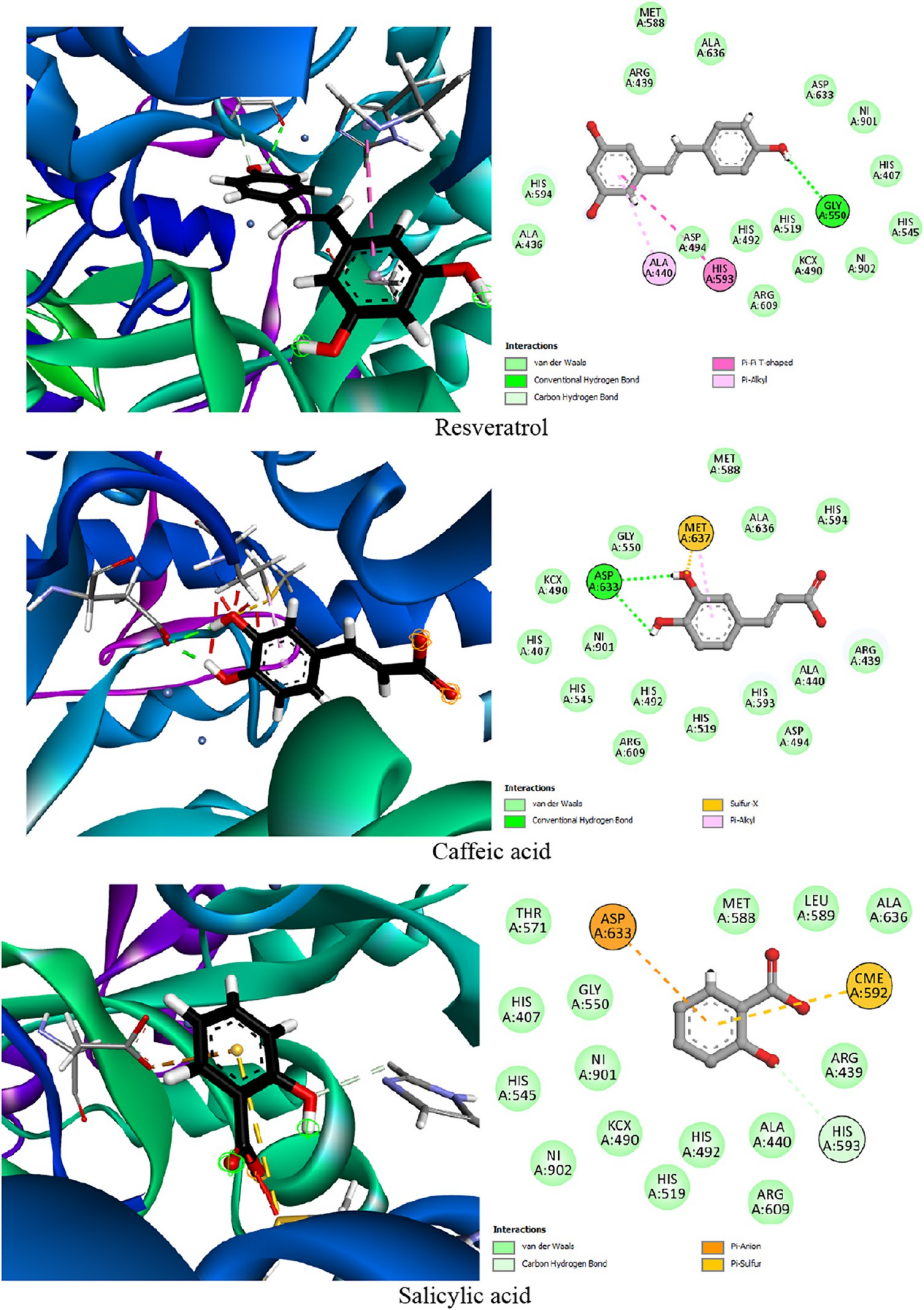
Two-dimensional (2D) and three-dimensional (3D) interaction diagrams of the top five *P. oceanica* phytocompounds within the active site of urease. The 2D illustrations highlight hydrogen bonds, hydrophobic contacts, and electrostatic interactions with key active site residues, while the 3D visualizations provide a spatial representation of the ligand-enzyme binding.

**Table 5:** Best Five Results for the Docking of *P. oceanica* Phenolic Ligands with Urease Target

compound	binding energy (kcal/mol)	hydrogen interactions (distance Å)	hydrophobic interactions	electrostatic interaction
thiourea	–5.1154	Ala440 (2.33), His593 (2.40), Asp633 (2.34), Kcx490 (2.56)	-	Met637
vanillin	–5.235	Arg609 (2.80), His519 (2.72), Asp494 (2.43), Arg439 (2.83), Asp633 (1.64) Ala636 (2.03)	Met637	Met637
hydroxybenzaldehyde	–5.158	Asp494 (2.71), Arg439 (2.17), Arg439 (2.69)	His593, Ala440	Asp633
ferulic acid	–4.825	His594 (2.77), Asp633 (2.66), Asp633 (2.45), Gly550 (2.54), His519 (2.72)	His409, His492	Arg439, Arg609, Met637
resveratrol	–4.784	Gly550 (2.47), Gly2.82 (2.82)	Ala440, His593	-
caffeic acid	–4.652	Asp633 (2.35), Asp633 (2.40)	Met637	Met637
salicylic acid	–4.362	His593 (2.62)	-	Asp633, Cme592

The docking results reveal that the phenolic compounds demonstrated varying degrees of binding affinities toward the urease active site, as evidenced by their binding energies and interactions with key residues. Among the tested ligands, vanillin exhibited the best binding affinity, with a binding energy of −5.235 kcal/mol, surpassing that of the reference inhibitor thiourea (−5.1154 kcal/mol). Vanillin formed multiple hydrogen bonds with active-site residues, including Arg609, His519, Asp494, Arg439, and Asp633, with notable interactions such as the short hydrogen bond with Asp633 (1.64 Å). Additionally, hydrophobic and electrostatic interactions involving Met637 further stabilized the binding of vanillin, emphasizing its potential as a strong urease inhibitor.

Hydroxybenzaldehyde ranked second in terms of binding affinity (−5.158 kcal/mol) and established hydrogen bonds with residues Asp494, Arg439, and hydrophobic interactions involving His593 and Ala440. Electrostatic interactions with Asp633 also contributed to its binding. This compound’s interaction profile highlights its effective engagement with essential residues in the urease active site, although its binding energy was slightly lower than that of vanillin.

Ferulic acid displayed a binding energy of −4.825 kcal/mol and formed hydrogen bonds with several residues, including His594, Asp633, Gly550, and His519. Ferulic acid’s binding was further supported by hydrophobic interactions with His409 and His492, and electrostatic interactions with Arg439, Arg609, and Met637. These interactions highlight the capacity of ferulic acid to establish a stable complex with urease, though its binding energy suggests moderate inhibitory potential.

Resveratrol and caffeic acid showed binding energies of −4.784 and −4.652 kcal/mol, respectively. Resveratrol formed hydrogen bonds with Gly550 and Gly (2.82 Å), while caffeic acid interacted with Asp633 twice through hydrogen bonds and engaged in hydrophobic and electrostatic interactions with Met637. These results suggest that while these compounds have lower binding affinities compared to vanillin and hydroxybenzaldehyde, they still exhibit notable inhibitory interactions.

Finally, salicylic acid demonstrated the lowest binding energy (−4.362 kcal/mol) among the tested compounds. It formed a single hydrogen bond with His593 and electrostatic interactions with Asp633 and Cme592, indicating weaker overall interactions with the urease active site compared to the other compounds.

The docking analysis highlights the critical role of specific residues in the urease active site, such as Asp633, Arg439, His593, Met637, and Ala440, which were frequently involved in hydrogen bonding and other stabilizing interactions. Notably, all tested ligands interacted with at least one of these key residues, similar to thiourea. However, the superior binding energy of vanillin indicates that it could potentially outperform thiourea as a urease inhibitor under certain conditions.

Interestingly, protocatechuic acid, although not ranked among the top-scoring ligands in our docking study, has been reported to display moderate inhibitory activity against urease in experimental assays. This apparent discrepancy between computational and experimental outcomes could be explained by several factors. First, the high polarity of protocatechuic acid may enhance its aqueous solubility and bioavailability, thereby facilitating effective interaction with the enzyme in vitro despite weaker predicted binding energy. Second, possible tautomeric equilibria of the catechol moiety may generate alternative binding conformations that were not fully captured by the docking simulations. Finally, synergistic effects with other phenolic metabolites present in complex plant extracts could also contribute to its observed biological activity. Taken together, these considerations highlight that docking results, while valuable for identifying binding trends, should be interpreted in conjunction with experimental data to fully assess inhibitory potential.

## Experimental Section

### Plant Materials

This study was conducted in compliance with all relevant institutional and international guidelines. Fresh *Posidonia oceanica* samples were collected from two distinct sites along Algeria’s northeastern coast: Stora, Skikda (36°53′N, 6°54′E; Figure 1A) in November 2022 (sample code: S.N), and La Caroube, Annaba (36°54′N, 7°46′E; Figure 1B) in July 2022 (C.J) and November 2022 (S.N) (Figure 5). All samples were collected at a depth of 8–10 m. All specimens were collected via free-diving before sunrise to minimize physiological stress, immediately placed in sterile bags containing in situ seawater with ice cooling (4 °C), and transported to the laboratory within 4 h for processing.

**Figure 5:**
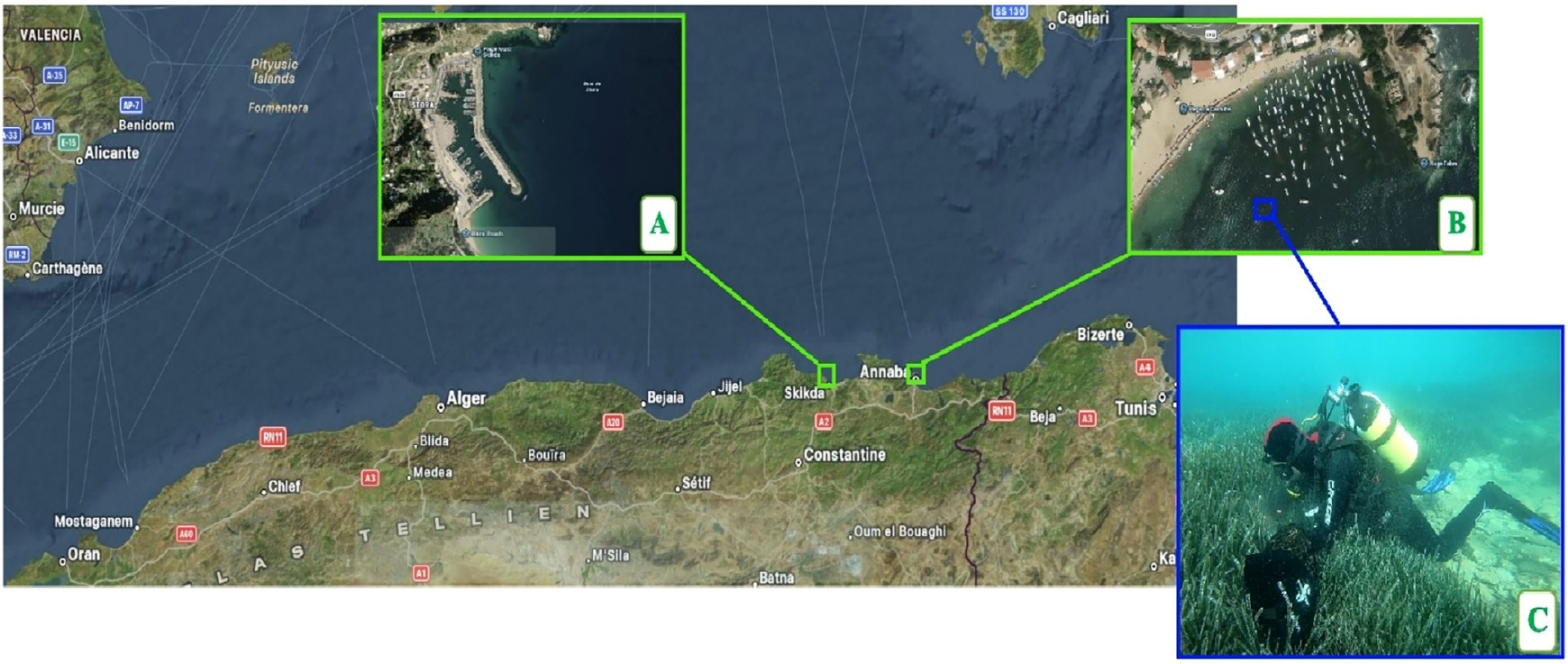
Sampling Sites of *P. oceanica* along the Eastern Algerian Coastline. The map shows the eastern coast of Algeria with the sampling sites clearly marked. (A) corresponds to Site 1: Stora, Skikda. (B) corresponds to Site 2: LA CAROUBE, Annaba. (C) shows a diver collecting samples of Posidonia oceanica at depths ranging between 8 and 10 m underwater.

### Extraction Methods

The leaves (F) and rhizomes (R) of *P. oceanica* were carefully separated and thoroughly washed with tap water to remove debris, epiphytic organisms, and other marine contaminants. A final rinse with distilled water was performed to ensure sample purity. The cleaned materials were then left to dry at room temperature until completely dehydrated. Once dried, the samples were ground into a fine powder using a laboratory grinder and stored in airtight containers at 4 °C until extraction.

Extraction was performed separately for the leaf and rhizome powders using the maceration method at a 1:5 (w/v) ratio with a methanol–water mixture (80:20, v/v), in the dark, at room temperature (22–25 °C), and under mechanical agitation for three consecutive 24-h cycles. In each cycle, the plant material was soaked for 24 h, followed by filtration using Whatman No. 1 filter paper, and then the residue was subjected to the next 24-h maceration cycle.^98^ After completing all three cycles, an additional filtration step using a low-pressure filtration system to enhance extract clarity. The solvents were then evaporated using a rotary evaporator at 35 °C. The resulting crude extracts were stored at 4 °C until further analytical and experimental procedures.^99^ Extraction yield of all extracts was calculated using the following equation below: Total extract yield, YT (%) = (Total mass of extract/Total mass of sample) × 100.

### Liquid Chromatography–Mass Spectrometry (LC-MS/MS)

Identification and Quantitative analysis of phenolics was carried out by LC-MS/MS (Agilent Technologies 1260 Infinity II, 6460 Triple Quad Mass spectrometer). Poroshell 120 SB-C18 (3.0 × 100 mm, I.D., 2.7 μm) column was employed.^100^ The mobile phase consisted of water (0.1% formic acid and 5 mM ammonium formate) as phase A and methanol (0.1% formic acid and 5 mM ammonium formate) as phase B, based on the protocol of Erenler et al, to ensure optimal ionization efficiency and accurate compound identification. Samples having 50 mg were transferred into eppendorfs having 2 mL. 2 mL ethanol were added to the resulting solution and stirred. The formed mixtre were extracted using hexane. Thereafter, the last mixture was centrifuged for and stirred at 9000 rpm 10 min. 100 μL sample was taken from methanol phases in the resulting solution and diluted to 900 μL as 450­(water)/450­(methanol). At the end the resulting last sample was filtered and analyzed by LC/MS-MS operating at 5.12 mL injection volume, 0.400 mL/min flow rate, 30.00 min method time and at 40 °C. Content analysis was performed based on 41 standard phenolic compounds.

### Activity Enzymatic

#### Anticholinesterase Activity

The inhibitory activity of acetylcholinesterase (AChE) and butyrylcholinesterase (BChE) was evaluated using Ellman’s colorimetric method in 96-well microplates.^101^ The assay measured the enzymatic hydrolysis of acetylthiocholine/butyrylthiocholine to thiocholine, which reacts with DTNB to produce a yellow-colored product detectable at 412 nm. The 200 μL reaction mixture contained sodium phosphate buffer (pH 8.0), enzyme (0.45 U/mL), the test solution containing *P. oceanica* extracts at different concentrations, DTNB (0.03 mM), and substrate (0.68 mM). Galantamine hydrobromide served as the positive control. The percentage inhibition was calculated using the formula: *I*% = [(*A*_c_–*A*_s_)/*A*_c_] × 100, where *A*_c_ and *A*_s_ represent the absorbance of control and test samples, respectively. IC_50_ values were determined from dose–response curves.

#### Urease Inhibitory Assay

The urease inhibitory activity of the *P. oceanica* extracts was determined based on the indophenol colorimetric method, as described by Taha et al.,^102^ with slight modifications. The assay was conducted in a 96-well microplate format. In each well, 25 μL of urease solution (5 U/milliliter of urease from Jack bean *Canavalia ensiformis*, type IX), 10 μL of the test sample (diluted in methanol at various concentrations), and 50 μL of urea solution (17 mM) were added. The reaction mixture was incubated at 30 °C for 15 min to allow for enzymatic hydrolysis of urea and subsequent ammonia release.

Following incubation, 45 μL of phenol reagent (8% w/v phenol and 0.1% w/v sodium nitroprusside) and 70 μL of alkaline reagent (2.85% NaOH and 4.7% available chlorine from NaOCl) were added to initiate color development. The plate was then incubated for an additional 50 min at room temperature.

The absorbance of the resulting indophenol blue complex was measured at 630 nm using a multimode microplate reader (PerkinElmer, Singapore). Thiourea was used as the standard urease inhibitor and served as a positive control. The percentage of urease inhibition and the IC_50_ values were calculated using the same methodology described previously for cholinesterase inhibition, based on absorbance differences between control and test samples and nonlinear regression of dose–response curves.

### Cytotoxicity Assay

#### Cell Line

The cell line used in this study is the Vero cell line, a continuous cell line derived from fibroblastic kidney cells of the African green monkey.

#### Cell Culture

Vero cells are cultured in RPMI medium supplemented with 5% fetal bovine serum (FBS) and a 1× mixture of antibiotics and antifungals. The cells are incubated in 25 cm^3^ culture flasks at 37 °C in a humidified atmosphere containing 5% CO_2_. Once the cells reach confluency, trypsinization is carried out. RPMI medium containing 2% FBS is then added to resuspend the cells, which are subsequently seeded into flat-bottomed 96-well plates.

#### MTT-Based Cytotoxicity Assay

The cytotoxicity assay performed is based on a colorimetric method using the MTT reagent, as described by Mosmann.^103^ This yellow compound is converted by a mitochondrial enzyme, succinate dehydrogenase, into a purple-colored derivative known as formazan. In the presence of MTT, wells containing viable cells turn purple, whereas those with nonviable cells remain yellow.

Vero cells, a continuous cell line derived from African green monkey kidney, were cultured in RPMI medium supplemented with 5% fetal bovine serum (FBS) and 1X antibiotic–antimycotic solution, and incubated at 37 °C in a humidified atmosphere with 5% CO_2_ using T-25 culture flasks. The passage number of the cells at the time of the assay was 122, and trypsinization was performed once confluence was reached. For the cytotoxicity assay, cells were seeded in flat-bottom 96-well plates using RPMI containing 2% FBS prior to treatment with the extracts.

The MTT cytotoxicity assay involves applying a serial 2-fold dilution (100 μL) of a plant extract onto confluent Vero cells in 96-well plates. The initial concentration of the extract is 2.5 mg/mL, which corresponds to an ethanol concentration of 3.75%—a level that is nontoxic to the cells. After 72 h of incubation, the dilutions are removed and replaced with MTT solution (100 μL). Following a 3-h incubation, the MTT solution is replaced by DMSO to solubilize the formazan (15 min contact time). The plate is then placed in a microplate reader, and the optical density (OD) of each well is measured at a wavelength of 630 nm, compared to the cell control.

The cytotoxicity of an extract is evaluated by determining its 50% cytotoxic concentration (CC_50_), which corresponds to the concentration that causes 50% inhibition of cell viability. The CC_50_ is defined as the concentration of the extract that yields an OD equal to half of that of the untreated control cells. All experiments were performed in triplicate to ensure reproducibility and reliability.

### Docking Molecular Analysis

An *in silico* docking analysis was conducted on THE 24 phytocompounds identified in the leaves and rhizome of *P. oceanica*. The study aimed to evaluate their inhibitory potential against three key enzymes: acetylcholinesterase (AChE), butyrylcholinesterase (BChE) and urease. Molecular docking simulations were performed using MOE 2015.10, beginning with the retrieval of the SMILES notation for each compound from the PubChem database, as previously described in the work of Benslama et al.^104^ The SMILES were converted into 3D structures, which were subsequently subjected to energy minimization to achieve stable conformations, ensuring optimal ligand geometry for docking. Receptor preparation involved multiple steps to optimize the structures of the enzymes for accurate docking predictions. The crystal structures of AChE (PDB ID: 4EY6), BChE (PDB ID: 4TPK), and urease (PDB ID: 4H9M) were downloaded from the Protein Data Bank. Water molecules and irrelevant heteroatoms were removed, while missing hydrogen atoms were added to the protein structures to ensure proper bonding. The protonation states of the residues were adjusted based on the physiological pH, and partial charges were assigned to reflect accurate electrostatic interactions. Finally, the active site regions were defined either by identifying the cocrystallized ligand binding sites or, in the case of urease, by utilizing CASTp 3.0 and literature-reported key amino acid residues.

A preliminary validation step was performed to verify the reliability of the docking protocol. This involved redocking the cocrystallized ligands of AChE (galantamine) and BChE (3F9) into their respective active sites. The root means square deviation (RMSD) values obtained from this redocking process were 0.665 Å for AChE and 1.320 Å for BChE, indicating acceptable precision of the docking methodology. For urease, the active site was identified using CASTp 3.0, supplemented by insights from existing literature that highlighted key amino acid residues involved in its catalytic activity.

Following docking, the binding poses of the phytocompounds were ranked based on their binding energy scores. The five top-scoring compounds for each enzyme were further analyzed to investigate their interaction profiles within the active sites. This detailed analysis was conducted using Discovery Studio 2024, allowing visualization and characterization of the key interactions, such as hydrogen bonding, hydrophobic interactions, and π–π stacking, that contributed to the compounds’ binding affinities.

### Statistical Analysis

The results were presented as arithmetic means accompanied by standard deviations (mean ± SD), based on three independent measurements. The IC_50_ and CC_50_ values were calculated using linear regression analysis. Analysis of variance (ANOVA) was performed using XLSTAT, version 2025.1.2.1430, to assess the presence of statistically significant differences among groups. For post hoc comparisons between means, Tukey’s multiple comparison test was applied, and differences were considered statistically significant at *p* < 0.001.

## Conclusions

The findings of this study highlight a wide range of future research and application prospects across the medical, veterinary, agricultural, and environmental sectors. In silico analyses of phenolic compounds identified in *P. oceanica* revealed their strong potential as natural inhibitors of key enzymes such as acetylcholinesterase (AChE), butyrylcholinesterase (BChE), and urease. Molecular docking simulations showed that polydatin exhibited binding affinity comparable to galantamine for AChE, rutin emerged as a potent BChE inhibitor, and vanillin demonstrated stronger urease inhibition than the reference compound thiourea. These findings support the therapeutic potential of these compounds, especially in managing neurodegenerative diseases and urease-associated gastric infections. Complementary in vitro assays further confirmed a moderate cytotoxic profile on Vero cells, with the active concentrations being significantly lower than toxic thresholds—indicating a favorable safety margin for further development.

From a veterinary perspective, particularly in poultry production, urease inhibition may improve gut health by reducing ammonia production and limiting the growth of urease-producing pathogens. This effect not only supports better nutrient utilization but may also enhance poultry meat quality by minimizing off-odors and improving sensory and physicochemical attributes. Therefore, *P. oceanica* extracts could be explored as natural feed additives and antibiotic alternatives in livestock production systems. Agriculturally, their ability to suppress soil urease activity presents a promising approach to minimizing nitrogen losses from urea-based fertilizers, improving nutrient efficiency, and mitigating environmental pollution. Thes findings not only highlight the theoretical potential of *P. oceanica* extracts but also support their practical application: they could be formulated as natural feed additives in poultry farms to reduce ammonia emissions, or incorporated into fertilizer formulations as biocoatings or supplements to enhance nitrogen utilization and minimize environmental impact. Optimizing dosage, method of application, and frequency based on experimental conditions would be essential for effective field implementation. Environmentally and industrially, the diverse phenolic and flavonoid compounds identified offer potential for eco-friendly applications, particularly in microbial control systems. Altogether, these results justify further *in vivo* validation, mechanistic studies, and formulation development to unlock the full multidisciplinary potential of *P. oceanica* as a valuable natural resource for health, agriculture, and sustainable development.

## Data Availability

All the data in the article are available from the corresponding author upon reasonable request.
